# “I do lack peace, and I’ve run out of answers”: primary caregivers’ perspectives on social and behaviour problems in cerebral malaria survivors in Blantyre, Malawi

**DOI:** 10.1186/s12936-022-04142-5

**Published:** 2022-04-13

**Authors:** Savannah Karmen-Tuohy, Sebastian M. Mboma, John T. Langfitt, Rachel Brim, Melissa Gladstone, Terrie E. Taylor

**Affiliations:** 1 New York University Grossman School of Medicine, New York, NY USA; 2 Blantyre Malaria Project, Kamuzu University of Health Sciences, Blantyre, Malawi; 3 Department of Neurology, School of Medicine and Dentistry, University of Rochester, Rochester, NY USA; 4 Department of Osteopathic Medical Specialties, College of Osteopathic Medicine, Michigan State University, West Fee Hall, 909 Wilson Road, Room B305, East Lansing, MI 48824 USA; 5 Department of Women and Children’s Health, Institute of Life Course and Medical Sciences, University of Liverpool, Alder Hey Children’s NHS Foundation Trust, Liverpool, UK

**Keywords:** Cerebral malaria, Neurodisability, Caregiver perspectives, Rehabilitation, Malawi

## Abstract

**Background:**

Despite recent advances in treatment and prevention, the prevalence of cerebral malaria (CM) remains high globally, especially in children under 5 years old. As treatment improves, more children will survive episodes of CM with lasting neurodisabilities, such as social and behavioural issues. Behaviour problems in children who survive CM are poorly characterized, and the impact of caring for a child with post-CM behaviour issues has not been well-explored. Caregivers’ perceptions of and experiences with their child’s post-CM behaviour problems are reported here.

**Methods:**

Semi-structured interviews were conducted with 29 primary caregivers of children who survived CM with reported behaviour issues in Blantyre, Malawi. Interviews were conducted in Chicheŵa, audio-recorded, transcribed, and translated into English. Data were coded manually, utilizing inductive and deductive approaches. Identified codes were thematically analysed.

**Results:**

Post-CM behaviours reported include externalizing, aggressive behaviours and learning difficulties. Variable timescales for behaviour change onset were noted, and most caregivers reported some evolution of their child’s behaviour over time. Caregivers experienced a variety of emotions connected to their child’s behaviour and to reactions of family and community members. Caregivers who experienced discrimination were more likely to describe negative emotions tied to their child’s behaviour changes, compared to caregivers who experienced support.

**Conclusions:**

Caregiver perceptions of behaviour changes in post-CM survivors are variable, and caregiver experience is strongly impacted by family and community member responses. Future educational, rehabilitation, and support-based programmes should focus on the specific types of behaviour problems identified and the difficulties faced by caregivers and their communities.

## Background

The incidence of cerebral malaria (CM) remains relatively high in sub-Saharan Africa, with over 65% of CM cases occurring in children aged 5 years and younger [1] CM, clinically defined as a patient with *Plasmodium falciparum* parasitaemia and coma with no other obvious cause, has a fatality rate of 15% [2–4]. Approximately one-third to one-half of CM survivors develop neurodisabilities, including motor, language, and sensory deficits, epilepsy, and social and behaviour problems [5, 6]. This proportion is likely to increase, as improvements in diagnosis and clinical management increase survival rates [7–9]. Previous studies have focused on characterizing outcomes and identifying barriers to rehabilitation [10–12]. Investigations into the behavioural and social impact of these disabilities is limited and compounded by a lack of culturally appropriate diagnostic tools available for use in these settings [13, 14].

Caring for a child with neurodisability in a resource-limited setting has a large impact on the emotional and mental health of caregivers, many of whom lack resources for social support and struggle to achieve community acceptance of their children [15, 16]. Caregivers of children with intellectual disabilities are at high risk for depression and psychological distress [15, 17]. Lack of support networks, inadequate finances, and limited knowledge about their child’s condition all contribute significantly to difficulties expressed by caregivers [15, 16]. In Malawi, rehabilitation services are limited and disproportionately distributed, with the bulk of resources concentrated in urban areas. The majority focus on physical disabilities, and limited attention is given to neurodisabilities and the related social impact [18]. To date, little qualitative research has been conducted to explore the caregiver experience with regard to behaviour problems in this setting; previous studies in Malawi have investigated caregivers’ perspectives on caring for children with neurodisability due to a variety of aetiologies [12], as well as explored how caregivers view their child’s neurodevelopmental outcomes within an established international framework [11]. While these studies provide a foundation of knowledge about rehabilitation efforts in this area, the lack of data focused on behaviour-specific outcomes remains a barrier to successful implementation of behaviour-specific rehabilitation and support programmes in Malawi.

In this study, caregivers’ perceptions of social and behaviour problems in children who survived CM were explored in order to more fully characterize the nature and scope of these problems. Specifically, caregivers’ perceptions regarding the onset of behaviour problems, changes in these behaviours over time, the causes of these behaviour problems, and the social impact on caregivers’ families and communities were explored, in order to better understand the effects.

## Study setting

A subset of caregivers of CM survivors participating in an ongoing, longitudinal exposure-control study on Cognitive Outcomes and Psychiatric Symptoms (COPS) of CM [10] were interviewed. In this cohort, 35% of caregivers had reported onset of new behaviour problems one year after hospital discharge, the majority (80%) of which were disruptive behaviours, such as aggression, arguing, hyperactivity, and disobedience [6]. Given these findings, a qualitative study nested within the COPS cohort was developed to further explore post-CM behaviours present in these children.

## Methods

### Study design, population and sampling

An exploratory qualitative research design was chosen to maximize the capacity to capture the large spectrum of post-CM behaviour problems that exist, many of which have not been well-described or investigated in the current literature. In addition, the available quantitative behaviour assessment tools are created for Western settings and are not validated in Malawi; a qualitative design was deemed more appropriate for exploring the depth and breadth of post-CM behaviour problems in this setting.

The intended study population was adult primary caregivers of CM survivors with behaviour problems. Caregivers of children in the COPS cohort were eligible to participate if they (a) were aged 18 years and above, (b) lived in Blantyre District, (c) reported new behaviours in their child at the one-, six- or twelve-month follow-up assessment, as identified by the question: “Do you have any concerns about your child’s behaviour since the last visit?” and (d) consented to participation in the study. Non-probabilistic purposive sampling was used to select caregivers who met the eligibility criteria of the study [19]. Out of 135 caregivers of CM survivors in the COPS cohort, 60 met eligibility criteria. Participants were initially contacted by SM (Programme Manager for the COPS study) in their households to gauge interest in the study and seek verbal consent to participate.

### Data collection

Data were collected via individual, semi-structured interviews to ensure confidentiality and to encourage respondents to speak openly without interruptions [19]. All interviews were conducted by SM, a fluent speaker of the vernacular language, Chicheŵa, with experience conducting qualitative interviews. SKT was also present during all interviews. The interview guide (Box 1) was piloted in advance, and adaptations to both translation of questions and unclear content areas were made. The guide focused on characterizing types of and changes in behaviour patterns and capturing the response of non-caregivers to behaviours. Transcripts were reviewed throughout data collection to ensure clarity of the questions and relevance of the data collected to the research objectives. After completing 29 interviews, the research team agreed that data saturation had been reached, based on the lack of both new themes discussed by interviewees and new codes emerging from analysis and review of transcripts throughout the interview process [20]. After this point, no further interviews were conducted. All interviews were conducted in Chicheŵa, audio-recorded, then transcribed in Chicheŵa and translated into English. Interviews were done in a private office at Queen Elizabeth Central Hospital.

Box 1: Guide for semi-structured interviews with caregivers of children with post-CM behaviour problems Caregiver perception

1. How long after your child was discharged from the hospital did you start to notice behaviour problems?
2. Share with us any other events, illnesses, or changes for your family that happened around the time the problems started.
3. Share with us some examples of what these problems were like when they started.
4. How did you respond when you started noticing these behaviours?
5. What do you think might have caused these behaviour problems to start?
6. Tell us what behaviour problems you still notice in your child.
7. Share with us the changes you’ve noticed in these problems, since they first started.
  - (probe): When did you notice these changes?
8. Have they been getting better or worse or have they stayed the same?
  - (probe): Tell us if there are things that have happened or things that you have done that you think made them better/worse?
9. Is there anything you would do differently to change < Child > ’s problems?

Family perception

10) What was your family’s perception of these problems?
11) How did members of your family respond to these problems?
12) What do members of your family believe caused these problems?
  - (probe): How have other people spoken about/acted on changes in these problems, since they first started?

Community perception

13) How did neighbours/members of the community perceive these problems?
14) How did neighbours/members of the community respond to these problems?
15) What do neighbours/members of the community believe caused these problems?
  - (probe): How have other people spoken about/acted on changes in these problems, since they first started?

Social impact

16) How did/have these behaviours affected < Child > ’s relationships with their [*ask separately*]
  - parents, grandparents, aunts and uncles,
  - brothers and sisters
  - friends
17) How have these behaviours limited < Child > ’s participation in
  - [*for children* > *5 years*] school
  - community activities (festivities, religious or other activities)

### Data management, quality assurance, and analysis

At the end of each interview, researchers cross-checked the accuracy of information collected through study participants’ reflections on their key responses as an informal method of member-checking [19]. Recordings were transcribed by one transcriber and translated by another to avoid bias or familiarity with the data. SM checked transcripts for quality both before and after translation by comparing randomly selected sections of audio, transcript, and translation; if any discrepancies were identified, they were discussed by the research team and corrected. Recordings, transcripts, and translations were de-identified and corresponding alphanumerical identifiers were assigned to each participant. Coding was done manually and independently by two researchers (SKT and SM), and comparison consensus was reached for the final set of codes. Both deductive and inductive approaches were used for coding. Researchers allowed the topic guide to inform a base set of themes, and these were supplemented with concepts emerging organically from the interviews.

## Results

Between May and July 2018, 29 interviews were conducted. Caregivers’ ages ranged from 27 to 64 years. Twenty-two were mothers, three were fathers, two were grandmothers, and two were sisters of CM survivors. Age range for CM survivors was between 6 and 15 years of age at the time of interviews. Six main themes emerged from the data.

### Variable times of behaviour onset

Caregivers identified a wide range of time-periods during which they perceived behaviour problems emerging, and some had difficulty being precise about the time frame. Some noticed problems as soon as their child was discharged from the hospital: “as soon as he recovered and he has started walking, it’s when this started” (BPS23). For others, the timescale was much longer. Five caregivers first noticed a change in their child one year or more after discharge; “Yes…2 years have gone now” (BPS4).

Caregivers recalled that some children were unable to walk, speak, or see for months after they returned home. In these children, caregivers perceived behaviour problems emerging when the child was well enough to return to school and resume playing with friends; “I saw that when she was with friends previously, she was playing with them well, but now I see some changes. She no longer plays with her friends well” (BPS4).

### Descriptions of behaviour problems

Using inductive analysis, caregiver perceptions of behaviour issues were broadly grouped into externalizing behaviours and learning difficulties.

#### Externalizing behaviours

The majority of caregivers perceived at least one externalizing behaviour in their child. The most commonly described behaviours were fighting with peers/siblings, using obscene language, short-temperedness, stubbornness, difficulty with directions, throwing stones, and destroying items. The number of caregivers who perceived each type of behaviour problem is presented in Table 1.

“When she is in conflict with a friend, whosoever responds, she stops the fight with this one and goes to that other one…So it doesn’t matter how many people respond, but she attempts to fight with them all.” (BPS5).

“She can’t stay at a place without fighting. She will make sure that she has fought someone. Someone has to cry.” (BPS27)

**Table 1:** Number of children exhibiting different externalizing behaviour problems, as perceived by caregivers

Behaviour problem	Illustrative quotes	Number of caregivers who perceived this behaviour in their child (n = 29)
Fighting with peers/siblings	“When he is amongst friends or relatives obviously one has to cry, and I know that he is the cause.” (BPS25)	24
Stubbornness/difficulty following directions	“When you go there to pick her, she will say ‘Leave me alone, you have to do your things and I will do mine.’” (BPS24)	18
Short-temperedness	“He takes time to forget things, maybe he can even refuse the thing because you have delayed giving it to him. Yes, he is quick tempered." (BPS19)	9
Throwing stones/stoning others	“My house is on top and there are other houses downwards, so he will just be throwing stones to those houses.” (BPS10)	5
Destroying items	“I don’t know what moves in his mind, recently he has broken the charcoal stove, it’s new.” (BPS23)	5
Using obscene language	“The main problem that was troubling me was fighting with his friends and swearing at old people.” (BPS1)	4

Some caregivers recounted examples of aggressive behaviour towards peers, which often occurred at school where teachers were unprepared to address the issue.

“There was this other time at school he stoned this other child. Then [father of the stoned child] chased him for a long distance, with a panga knife in his hand running after him that he should chop him.” (BPS23).

In many cases, caregivers shared that peers and friends were often confused after experiencing unsolicited aggression from their child, recalling responses like: “…what have we done to wrong you?” (BPS8).

#### Learning difficulties

The majority of caregivers perceived some degree of memory problems in their children. For some children, these problems were most noticeable at home, as their behaviour disrupted the family’s daily routine.

“At times she would ask ‘Mother, what relish are we having today?’ I would answer ‘Cabbage,’ then she would come again and ask the same question and I would shout at her to say, ‘Haven’t I already answered you on that one, we are going to have cabbage.’” (BPS9).

“You can ask him to go inside the house to get something. He will go, but he will bring a different thing then what you have asked him to get. Even if you sent him to the shop, he will buy things which you didn’t tell him to buy.” (BPS23).

Other caregivers recalled being alerted to memory problems due to their child’s poor performance or lack of attendance in school, and many worried about their child failing their grade level multiple times.

“When he could go to school, he could be entering other classes. When you ask him why, he would say ‘I didn’t know this is not my class, I thought it was the usual class I attend daily.’” (BPS10).

“My daughter is having problems at school because the friends she was born in the same year with are now going into Standard 5, while she is [still] in Standard 2.” (BPS2).

### Perceptions of the causes of behaviour problems

All but one caregiver perceived their child’s episode of CM as the cause of the behaviour issues. The majority of caregivers demonstrated a clear understanding of the effect of CM, even when they did not expect the illness to manifest itself in behavioural issues.

“I feel it’s just an illness that attacked her, [it is] the impact of malaria, but I never imagined that she would reach this extent.” (BPS5)

One caregiver shared a belief that the behaviour problems were due to residual illness or untreated symptoms:

“I feel that maybe the illness is still there, or that it’s starting again, and that’s why the child is showing this behavior, or maybe the dosage that the doctors gave him was not complete, or he wasn’t completely healed.” (BPS3)

### Changes in behaviour over time

Most caregivers perceived that their child’s behaviour improved over time. Even with this improvement, most caregivers said some behaviour issues were still present, though much less extreme; “I know the conscience has started coming because she is growing up, and the negative thoughts are fading…she can’t just change overnight” (BPS2).

A small number of caregivers perceived their child’s behaviour problems as worsening; “The issue is that of school progress, it’s getting worse… When he goes to school, he doesn’t attend classes. He was a very hard-working child. He was doing well, but, as of now, I am doubtful if he can pass. I don’t see any aspect of school in him” (BPS28).

### Caregiver emotions concerning behaviour changes

In each interview, the researchers sought to identify caregivers’ perceptions of the emotions tied to their child’s behaviour problems. Four main expressions emerged from the transcripts. Twelve of the 29 caregivers expressed mainly negative emotions surrounding their child’s behaviour problems, while eight caregivers expressed mainly positive emotions. The remaining caregivers reported a spectrum of positive and negative emotions.

#### Worry and “lacking peace”

Multiple caregivers described a sense of “lacking peace” about their child’s condition; “When he is fighting with friends, people around here even hate me, even his relatives…I do lack peace and I’ve run out of answers” (BPS1).

Many caregivers experienced a sense of worry about the negative impact of their child’s behaviour on their tightly knit community, where any small mishap could lead to exclusion of the child and stigmatization of the entire family.

#### Shame

Most caregivers lived in close communities, and many shared feelings of shame about their child’s behaviour when it was disrespectful toward others.

“When she does something wrong, I rush there to ask for forgiveness. When she comes infuriated, I rush to say sorry.” (BPS9)

Many caregivers spoke about the added burden of not only caring for their child, but also defending their child in the community, describing the experience of constantly being prepared to mitigate disputes resulting from their child’s actions; “You get worried that if a visitor may come, you will be ashamed” (BPS1).

#### Acceptance

Some caregivers experienced feelings of acceptance; “There are other children whose abnormalities are visible, so sometimes we just accept that his abnormality is invisible” (BPS19). Caregivers spoke about the responsibility that comes with having a child with a disability; “I welcomed it as a parent seeing that [my daughter] is mine, and I can’t leave her with anyone else” (BPS2).

Other caregivers shared that they had been able to accept the behaviour problems after fearing that their child would die during hospitalization. Caregivers perceived surviving malaria with some impairment to be a much better outcome than dying from the disease; “I receive it, and I was very proud. Because I didn’t expect that my child will come out of the hospital alive…I receive it because even if I can have money and go to the market, I will not be able to buy the life of my child” (BPS13).

When caregivers were asked if they would have done anything differently to avoid their child getting sick, most caregivers did not believe that there was anything else they could have done. There was a sense of acceptance about CM among many of the caregivers, with one grandmother stating: “These [diseases] just come out of the blue. It just comes any time. You just accept it that it’s like this” (BPS23).

#### Despair

Caregivers were very vocal about their experiences with the limited support available to cope with their child’s behaviour changes. Many caregivers perceived the COPS study team and the scheduled 6-month follow-ups as their only resource.

“When we came to the hospital during the last visit, we were told that his time has come to drop out from the research and that’s why he has been given this certificate. So, it was up to me to think where will I go with him with this stubbornness since those people are done with him…I had nowhere to go.” (BPS3)

“I can’t talk much but I can just appreciate the way you [COPS study] helped me when I came here to the hospital…This is the only place where I have been helped.” (BPS25)

### Caregiver experiences addressing behaviour problems

#### Stigmatization by community members

Caregivers who felt shame and despair about their child’s condition often reported experiencing negative interactions in their communities. Some caregivers perceived themselves as targets of stigmatization from their community, as people suspected witchcraft or HIV infection to be the cause of the child’s behaviour problems.

“They were saying that the child has been bewitched… But also, they said that the child has diseases, because the father is [HIV] positive. They said that this sickness, it’s the beginning of the disease.” (BPS11)

“The rumors I heard are…that some were saying that we wanted to perform rituals on the child or eat her and the like.” (BPS2)

Caregivers also perceived discrimination from members of their community in the form of name-calling their child. One caregiver shared,

“Instead of calling him by name they were calling him all sorts of names, instead of calling him [child’s name], they were calling him abnormal, I wasn’t happy with that.” (BPS10)

Others gave examples of specific names used for their child, all of which are severe and stigma-laden language in Chicheŵa.

“Others say ‘malungo ziii’ and others say ‘okufa nkudzuka.’” (BPS21)

“For his friends they don’t argue with him, they just say that he is ‘kadzifere’ so don’t bother arguing with him…They gave him that name.” (BPS28)

“*Malungo ziii*” is a reference to a public health campaign aimed at total prevention of malaria in Malaŵi. In using this language, caregivers believed that community members were suggesting that the child “belonged” to that outreach campaign, because he suffered from CM. Both “*okufa nkudzuka*” and “*kadzifere*” can be interpreted as “with a fragile life.” In context, these names were used for children who lost consciousness during CM infection and who were subsequently viewed as dying and coming alive again. The sentiment, as perceived by caregivers, was that these children could easily die, and, therefore, were viewed as less productive members of the community.

As perceived by caregivers, relatives and neighbours often blamed caregivers for not disciplining their child enough or “pampering” their child at home. Caregivers felt that they were often put in a position to defend the actions of their child and explain that more discipline would not change their child’s behaviours.

“When people see her, they say that I am the one who caused that, because I pampered her… They say that I pampered the child when she was young.” (BPS9)

“I feel pain because what happens to me as a parent, even if I discipline her, the onlookers think I don’t do anything in terms of my child.” (BPS2)

“When I could explain to [my relatives], they would say that just discipline her we shouldn’t be in conflict with people in this village.” (BPS5)

#### Acceptance from community members

Caregivers who conveyed a level of acceptance about their child’s behaviour problems often identified supportive family and community members, who understood CM to be the cause of the behaviour problems. In most cases, caregivers perceived this support coming from relatives or neighbours who had previously encountered another child with similar behaviours after malaria infection.

“Most people around are getting used to him, because there was also another one who has the same problem, so they know this from that one.” (BPS19)

Caregivers perceived receiving the strongest support when they shared information about their child’s illness with community members, specifically those who frequently interacted with their child. Once made aware, community members provided additional attention and support to the child, often leading to improvements in behaviour patterns.

“At first, she wasn’t performing well, so I went to her teacher because she repeated Standard 1, so I went to her teacher to say the child is not passing well so you should pay attention to her…That’s when I saw that she passed and went to Standard 2.” (BPS6)

“I even told his teacher about this problem…and he was like ‘Why didn’t you tell us all this time, we have to know now.’ So even if he is wrong, he is well protected.” (BPS23)

#### Effect on school and community involvement

Many caregivers perceived their child’s behaviour problems as having a large impact on their performance in school and engagement in community activities. In addition to learning difficulties, a majority of caregivers reported that their child had lost interest in regularly attending school.

“Sometimes maybe when he is on break, he will knock off. We know him by face that he has done something wrong, like knocking off during break time, as he will be walking slowly coming home. But if he has knocked off on time, he will come home running.” (BPS11)

“I have noticed that he doesn’t go to school regularly. This is the third time repeating the same class. Sometimes he does [bring homework] and sometimes he tears it up.” (BPS23)

Caregivers also perceived a lack of interest in community activities in their children. Children often lacked desire to participate in church services and failed religious classes, echoing problems observed in the school setting.

“She doesn’t like going to church nowadays, but before she got sick, she used to sing, she was a member of the choir… When you tell her to go to church she will say ‘aaah I will go next week.’” (BPS12)

“There are these children’s classes from the age of 6. I registered him, first phase he failed, and he didn’t go again, he stopped. As I am speaking now, his friends will be confirmed this month of June. But with him, there is nothing about church in him.” (BPS28)

Caregivers also shared their perception of the effect of their child’s behaviour on their family as a whole. One caregiver (BPS9) stated that her daughter “loves those people she is not related to more than her relatives.” Caregivers recalled that their children fought often with their siblings, causing disruption in the household.

“They are always arguing unless I separate them. They don’t even like eating together – she keeps saying ‘He is unhygienic, I will eat myself.’” (BPS9)

“She was treating [her sisters] very bad. The moment I leave home she fights them. When I come back, I hear from the neighbours that ‘iii you have to talk to your child, otherwise you will find your other children being killed.” (BPS24)

A few caregivers experienced difficulty in dividing time and resources evenly between their children, because the post-CM child required so much attention. Overall, caregivers perceived that their lives were dramatically impacted by both their child’s behaviour and subsequent discrimination from members of the community and their family.

## Discussion

This study provides a richer understanding of behaviour problems that commonly occur among children who survive CM than has been previously available. Using qualitative methods to understand the perceptions of primary caregivers, a range of post-CM behaviour problems and their impact on daily life were identified. All but one caregiver described issues with externalizing behaviour in their child and fighting with peers/siblings was the primary behaviour issue. Past studies have categorized post-CM behaviour problems as externalizing or internalizing [21–23]. Ours was a qualitative exploratory study, rather than a quantitative study using diagnostic assessment tools, so internalizing behaviours may have been underreported. The open-ended nature of the questions and their focus on family and community impact may have led caregivers to focus on more overt, externalizing behaviours, rather than on more nuanced internalizing behaviours.

Half of the caregivers identified learning difficulties due to memory or attention problems in their children, consistent with studies of CM survivors in other African countries [23–25]. Many CM survivors have been found to exhibit deficits in multiple cognitive domains, including memory and attention, and ADHD-like symptoms have been the most frequently observed behaviour problem after CM in other cohorts [24, 25]. Persistent language deficits have also been observed in some cohorts [5, 26]. While issues with language were not specifically discussed by many caregivers in this study, communication impairment could play a role in the academic difficulties that emerged in multiple narratives.

All but one caregiver linked their child’s behaviour problems to their past episode of CM. This may over-estimate the average caregiver’s understanding of CM sequelae, as these caregivers acquired a level of health literacy about CM through COPS follow-up visits. Many caregivers pointed to involvement in the COPS study as a valuable resource for answers to general questions about their child’s condition and a venue to share their concerns.

Caregivers’ perceptions of behaviour onset varied substantially between participants, from onset at hospital discharge to multiple years post-CM. A greater proportion of caregivers who reported behaviour onset occurring within the first month after hospital discharge described learning difficulties in their children (7/12 caregivers), compared to caregivers reporting behaviour onset between one month and one year from discharge (1/3 caregivers) and caregivers reporting onset greater than 1 year after hospital discharge (2/5 caregivers). All but one caregiver, regardless of behaviour onset timeline, reported externalizing behaviours in their child emerging at some point. Birbeck et al. observed different onset time periods for neurological sequelae after CM, with motor, sensory, and language impairment emerging first, followed by behavioural issues and, lastly, epilepsy [5]. In a separate cohort, CM survivors had more externalizing behaviour problems than controls at 12-month follow-up, and more internalizing, externalizing, and total problems than controls at 24-month follow-up [22]. The current study is unique in that identification of behaviour problems was not limited to a specific follow-up schedule, and information about behaviour onset was based on the subjective perception of the caregivers, both of which likely explain the variety in responses gathered and why these data may deviate from prior findings.

While the majority of caregivers perceived their child’s behaviour as improving over time, a minority voiced that their child’s behaviour was unchanged or worse. Among caregivers whose child’s behaviour had worsened, it was most often in the context of one particular area of behaviour, while behaviours in other areas had resolved. Multiple caregivers shared that their child’s externalizing problems improved, but they were still struggling with academic tasks or memory problems: “That act of fighting is minimal now, but loss of memory is getting worse” (BPS23). Previous studies have involved a single time point assessment of neurodisability and behaviour; this study design provided an opportunity to address changes in behaviour over time [21, 23, 24]. Other studies involving multiple observations had shorter follow-up periods or used a quantitative approach and did not seek to characterize caregiver perceptions of behaviour change [5, 22, 27, 28]. Investigation of caregiver perceptions of behaviour change addresses the impact that the behaviour has on the family and community; children who score below a diagnostic threshold on a quantitative tool may still exhibit disruptive behaviour in their daily interactions with others.

Caregivers expressed a variety of emotions about their child’s disruptive behaviour, from despair and shame to acceptance and relief about their child’s survival. Caregivers who experienced negative emotions often reported stigmatization and a lack of acceptance from relatives and community members. Those caregivers who felt more positive emotions often attributed this to receiving support and understanding from their communities. Nakitende et al. describe similar themes emerging in interviews with Ugandan caregivers whose children experienced either a single episode of severe malaria or an episode of severe malaria followed by multiple repeated malaria infections [29]. Both studies highlight the emotional burden on caregivers of children who survive CM with neurodisability and the profound impact that caring for these children can have on family and community dynamics.

Caregivers’ experiences of post-CM behaviour problems in their children and their perceptions of the effect of these behaviours on daily life vary greatly. Further research should focus on better characterization of the types of behaviour issues exhibited and their time of onset, with the goal of creating more culturally appropriate, structured assessment tools for post-CM behaviours in this patient population. With additional information about caregiver perceptions of the impact of post-CM behaviours on their families and communities, future efforts can be directed toward creating and implementing support services and rehabilitation programs for this population.

### Limitations

The study was specifically designed to include the behaviour changes that were noted by the caregiver, rather than identified by team members through COPS standardized and validated assessment tools. Narratives of behaviour problems that were exclusively identified by the caregiver were sought, as the study aim was to understand behaviour changes from a caregiver perspective. Only those behaviours that actively impacted daily and community life were explored. Types of behaviour and onset time were based solely upon caregiver recollection. Descriptions of behaviours and timeframes, therefore, could have been limited by caregiver knowledge, memory, and language expression.

## Conclusions

Caregivers of post-CM survivors reported a variety of behaviour changes with different times of onset in their children, most frequently describing externalizing behaviours and learning difficulties. Caregivers expressed a wide spectrum of emotions associated with these behaviour changes, and family and community member responses had a large impact on caregivers' perceptions of their child’s behaviour. With a better understanding of the types of post-CM behaviour problems observed in children and the daily experience of their caregivers, future educational and rehabilitation interventions should target specific behaviour problems identified and aim to establish programs and outreach services to further support caregivers in their communities.

## Data Availability

The datasets analysed during the current study are available from the corresponding author on reasonable request.
